# Lower Extremity Joint Contributions to Trunk Control During Walking in Persons with Transtibial Amputation

**DOI:** 10.1038/s41598-019-47796-z

**Published:** 2019-08-22

**Authors:** Adam J. Yoder, Amy Silder, Shawn Farrokhi, Christopher L. Dearth, Brad D. Hendershot

**Affiliations:** 1 DoD-VA Extremity Trauma and Amputation Center of Excellence, Various locations, USA; 2 Department of Physical & Occupational Therapy, Naval Medical Center, San Diego, CA USA; 3 Department of Rehabilitation, Walter Reed National Military Medical Center, Bethesda, MD USA; 4 Department of Surgery, Uniformed Services University of the Health Sciences, Bethesda, MD USA; 5 Department of Rehabilitation Medicine, Uniformed Services University of the Health Sciences, Bethesda, MD USA

**Keywords:** Biomedical engineering, Biological physics, Trauma, Translational research

## Abstract

Controlled trunk motion is crucial for balance and stability during walking. Persons with lower extremity amputation often exhibit abnormal trunk motion, yet underlying mechanisms are not well understood nor have optimal clinical interventions been established. The aim of this work was to characterize associations between altered lower extremity joint moments and altered trunk dynamics in persons with unilateral, transtibial amputation (TTA). Full-body gait data were collected from 10 persons with TTA and 10 uninjured persons walking overground (~1.4 m/s). Experimentally-measured trunk angular accelerations were decomposed into constituent accelerations caused by net joint moments throughout the body using an induced acceleration analysis. Results showed persons with TTA had similar ankle moment magnitude relative to uninjured persons (*P* > 0.05), but greater trunk angular acceleration induced by the prosthetic ankle which acted to lean the trunk ipsilaterally (*P* = 0.003). Additionally, persons with TTA had a reduced knee extensor moment relative to uninjured persons (*P* < 0.001), resulting in lesser sagittal and frontal induced trunk angular accelerations (*P* < 0.001). These data indicate kinetic compensations at joints other than the lumbar and hip contribute to altered trunk dynamics in persons with a unilateral TTA. Findings may inform development of new clinical strategies to modify problematic trunk motion.

## Introduction

Persons with lower extremity amputation (LEA) often exhibit abnormal trunk motion during functional tasks relative to uninjured persons^1^. Altered trunk motion poses concern as there is evidence of association between altered trunk dynamics and prevalent, deleterious health conditions in persons with LEA^1^. For example, increased trunk flexion and flexion velocity during trip recovery have been associated with increased fall likelihood in persons with LEA^2^. Additionally, persons with a unilateral LEA often self-report perceiving “uneven posture and compensatory movements of the back” as a primary factor in chronic low back pain^3^. However, the underlying mechanisms by which persons with LEA modulate control of angular trunk dynamics are not well understood.

Dynamic walking simulations have proven useful to investigate mechanical phenomena difficult or impossible to test using experimental measurement^4^. Among uninjured persons, induced acceleration (IA) analyses have been used to decompose net trunk angular accelerations into constituent accelerations corresponding to net joint moments^5^, and underlying muscle forces^6^, throughout the whole body. Specifically, simulations suggest sagittal trunk motion is modulated primarily by sagittal joint moments, where the net lumbar extensor moment acts to rotate the trunk posteriorly during early stance, in opposition to extensor moments about the hip, knee, and ankle, which act to rotate the trunk anteriorly^6^. In contrast, control of frontal trunk motion appears bi-planar, where the frontal lumbar and stance limb hip abductor moments act to rotate the trunk contralaterally away from the stance limb, in opposition to sagittal moments about the stance limb hip, knee, and ankle, which act to rotate the trunk ipsilaterally^6^. These and similar IA simulations^7^, exemplify how muscles and net joint moments can act to induce counter-intuitive segment accelerations throughout the body.

Prior observational studies of persons with a unilateral, transtibial amputation (TTA) report an array of deviations in joint and muscular kinetics during walking relative to uninjured persons. During early stance, persons with unilateral TTA exhibit: a reduced affected limb knee extensor moment^8,9^, a reduced affected limb hip abductor moment^10^, and greater hip extensor moments and power generation in both the intact and affected limbs^11,12^. Greater trunk-pelvis lateral bend and sagittal extension moments^13^ have also been reported and are thought to be associated with greater muscular forces in back extensor and abdominal musculature^14^. Given that lower extremity net joint moments contribute to trunk angular accelerations in uninjured persons^5,6^, studies are warranted that assess how deviations in lower extremity moments contribute to altered trunk motion in persons with a unilateral TTA.

The aim of this study was to characterize how compensations in lower extremity joint moments contribute to altered trunk angular dynamics in persons with a unilateral TTA during walking. We expected lumbar and lower extremity net joint moment magnitudes in persons with a TTA would differ relative to uninjured persons, similar to prior reports^8,10,11,13^. Therefore, we hypothesized that trunk angular accelerations induced by the lumbar and lower extremity net joint moments would also differ between groups.

## Methods

### Experimental protocol

Ten male subjects with unilateral TTA wearing passive energy storage and return prosthetic feet, and ten male uninjured subjects were identified from records of the Biomechanics Lab at Walter Reed National Military Medical Center (Table 1). All subjects had provided written, informed consent under a protocol approved by the Institutional Review Board at the Walter Reed National Military Medical Center. All research was performed in accordance with relevant guidelines and regulations. Inclusion criteria for this retrospective analysis were a self-selected walking velocity within +/−5% of the combined sample mean and no current low back pain or comorbidities per self-report that would affect gait mechanics at time of data collection.

**Table 1:** Mean (standard deviation) demographics for subjects with transtibial amputation (TTA) and uninjured subjects.

Group	Age (year)*	Mass (kg)*	Height (cm)^*^	Time From Amputation (year)	Self-Selected Speed (meter/sec)*
Uninjured (n = 10)	31.3 (7.5)	89 (6)	183 (6)	—	1.44 (0.15)
TTA (n = 10)	30.0 (7.7)	87 (12)	179 (4)	1.5 (0.3)	1.38 (0.14)

All TTA subjects wore passive energy storage and return prosthetic feet (n = 3 Endolite Elite Blade, n = 3 Freedom Innovations Renegade MX, n = 1 Endolite Echelon, n = 1 Ossur Re-Flex VSP, n = 1 College Park Soleus, n = 1 WillowWood Pathfinder).

*No demographic parameters were different between groups (independent t-tests, all *P* > 0.05).

Subjects walked overground along a 15 meter walkway at their self-selected speed. Three-dimensional retro-reflective marker trajectories and ground reaction forces were simultaneously measured via a 27-camera motion capture system (Vicon, Oxford, UK) and six floor-embedded force platforms (AMTI, Watertown, MA, USA). Marker trajectories were sampled at 120 Hz and ground forces at 1200 Hz. For each subject, one representative stance phase was selected with three consecutive plate strikes: intact/prosthetic/intact for TTA, and left/right/left for uninjured.

### Modeling & simulation

A processing workflow was employed using the open-source OpenSim software v3.3^15^ and MATLAB API scripting for automation (MathWorks Inc, Natick, MA, USA). Marker trajectories and analog force data were lowpass filtered using a fourth order bi-directional Butterworth filter with cutoffs of 6 Hz and 25 Hz, respectively. The generic, Gait2392 model anthropometry was scaled in a static standing posture, using a consistent set of virtual-to-experimental marker pairs across all subjects. A global optimization inverse kinematics algorithm was used to compute joint angles by minimizing tracking error between model and experimental marker trajectories^16^. Joint angles and experimental ground reaction forces were input to the Residual Reduction Algorithm (RRA) to reduce dynamic inconsistencies between whole body model motion and measured ground forces^15^. Joint moments and adjusted joint kinematics were input to an IA analysis that calculated trunk angular accelerations resulting from application of each net joint moment in isolation. Linear superposition was verified for each subject - specifically, the net total summation of all constituent IA contributions from net joint moments was compared to the trunk segment angular accelerations measured experimentally. Ground reaction forces in the IA analysis were predicted using a rolling-without-slipping kinematic constraint at each foot^17^. Quality metrics were computed for inverse kinematics, RRA, IA superposition, and GRF constraint prediction, and assessed against recommended quality standards^18^ (Supplementary Tables S.2–S.5).

### Statistical analysis

Statistical parametric mapping (SPM) was used to test for differences between TTA and uninjured subjects^19^. Whereas standard statistical tests output a scalar test statistic, SPM outputs a test statistic trajectory that defines time durations over which compared one-dimensional trajectories differ between groups, as exemplified in Supplementary Fig. S.1. All group comparisons were done using an un-paired, two-tailed SPM t-test (α = 0.05), with normalcy verified using a Shapiro-Wilk test. Joint moment magnitude and the corresponding trunk IA trajectory were compared for the following: bilateral ankle plantar/dorsiflexion, knee flexion/extension, hip flexion/extension, hip ab/adduction, hip internal/external rotation, and the tri-planar trunk-pelvis moment. Trunk IA trajectories due to gravity and segment velocities ewere additionally compared. Lastly, experimentally-measured trunk segment angular acceleration, velocity, and angle with respect to a global reference were compared. For each statistically significant duration in an IA trajectory, the mean difference magnitude between groups was computed and verified to exceed cumulative IA caused by model residual forces and moments after RRA. This check ensured significant differences exceeded measurement and simulation uncertainties.

## Results

### Simulation verification & validation

**Figure 1:**
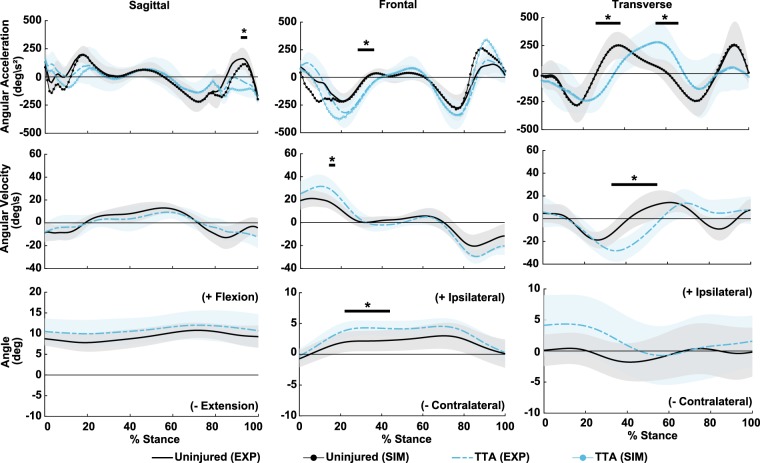
Ensemble average trunk segment angular kinematics relative to global for the transtibial (TTA) (dashed line) and the uninjured group (solid line). Shaded areas represent 1 sample standard deviation. Thick horizontal bars (*) indicate stance durations where trajectories were significantly different between TTA and uninjured groups. For each y-axis, positive signage indicates leaning/twisting towards ipsilateral (prosthetic) stance limb for frontal/transverse motion and leaning anterior for sagittal motion. A comparison of experimentally-measured (EXP, top row) trunk segment angular acceleration against the net summation of all accelerations induced by joint moments and gravity (SIM, top row) verified the assumption of linear superposition for the IA analysis.

Across all markers, inverse kinematic tracking differences during stance fell at or below 1.6 cm (1.1 cm root-mean-square average) (Supplementary Table S.2). Magnitudes of residual forces and moments applied to the pelvis after RRA were below 5.0% of the maximum measured external ground reaction force and below 1.0% maximum external force * body height for residual moments^18^ (Supplementary Table S.3). Ground reaction forces predicted by foot kinematic constraints closely reproduced experimentally measured forces; the largest disagreement was 2.2% of maximum external force in the vertical direction (Supplementary Table S.4). Lastly, the net total summation of all joint moment IAs (SIM, Fig. 1) compared well with experimentally-measured trunk segment angular acceleration (EXP, Fig. 1), which verified the linear superposition assumption (Supplementary Table S.5).

### Trunk segment kinematics

Several differences were identified in angular kinematics of the trunk for the TTA group relative to the uninjured group (Fig. 1, Supplementary Table S.1). In the frontal plane, the TTA group had greater ipsilateral lean over the affected limb during 22–44% stance (%ST) (*P* = 0.032), greater ipsilaterally-directed velocity (14–17%ST, *P* = 0.045), and greater ipsilateral acceleration (28–36%ST, *P* < 0.001). In the sagittal plane, flexion angle and flexion velocity trajectories were similar between groups, although a brief duration of lesser flexion-tending angular acceleration was measured in the TTA group during 92–95%ST (*P* = 0.025). In the transverse plane, trunk angle was similar between groups, while angular velocity (33–55%ST, *P* < 0.001) and acceleration differed (26–38%ST, *P* < 0.001, 55–66%ST, *P* = 0.001) (Fig. 1).

### Joint moments

**Figure 2:**
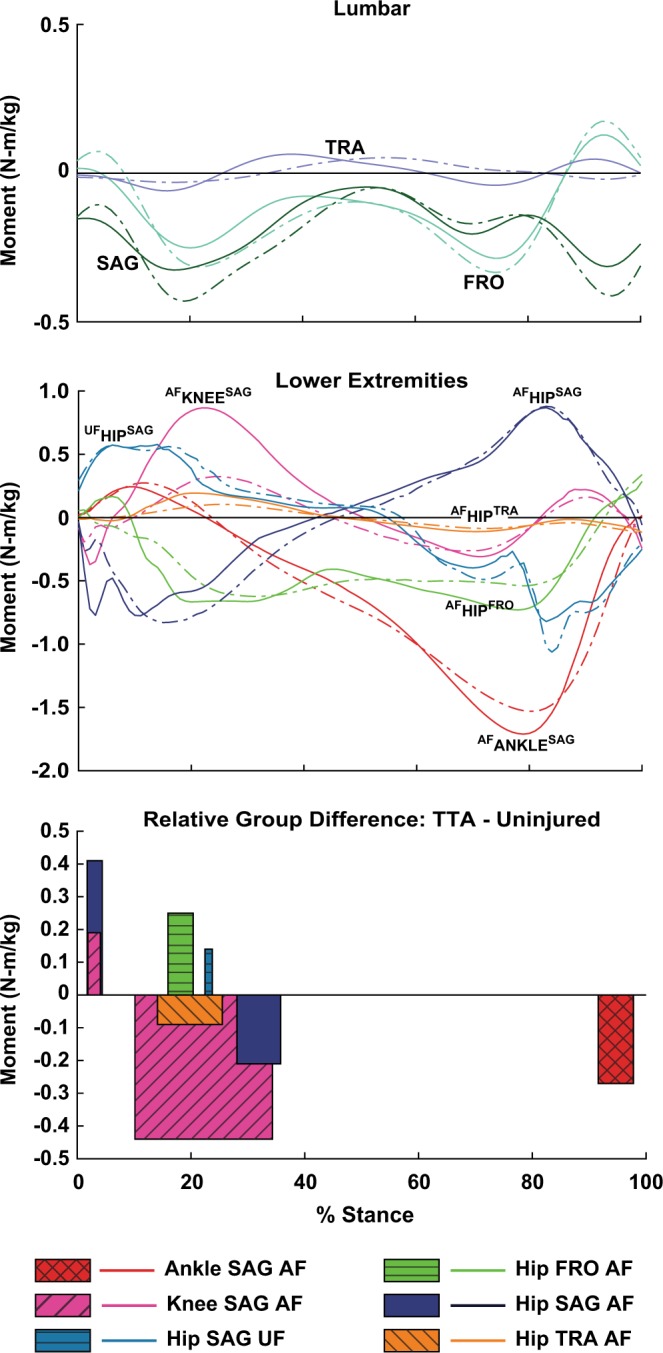
Ensemble average joint moment trajectories for the transtibial (TTA, dashed line) and uninjured group (solid line) in the lumbar (top) and affected stance limb (middle). Significant between group differences (bottom) are identified in duration by bar width and mean difference magnitude by bar height. A positive difference indicates the TTA group had a more positive moment over the duration, relative to the reference uninjured group. Note there were no significant differences in lumbar moment trajectories (top). AF = Affected limb, UF=Unaffected limb. Moment signage (internal): +ankle = dorsiflexor, +knee = extensor, +hip sagittal = flexor, +hip frontal = adductor, +hip rotation = internal, +lumbar SAG = flexion, +lumbar FRO = ipsilateral, +lumbar TRA = ipsilateral.

The prosthetic ankle plantarflexor moment was greater during 92–98%ST in the TTA group relative to the uninjured group (*P* = 0.002, Fig. 2, Supplementary Table S.1). Additionally, the affected limb sagittal knee extensor moment was lesser during 10–34%ST (*P* < 0.001), the affected limb sagittal hip extensor moment was greater during 28–36%ST (*P* = 0.003), and the affected limb hip abductor moment was lesser during 16–20%ST (*P* = 0.017).

### Trunk angular accelerations induced by joint moments

**Figure 3:**
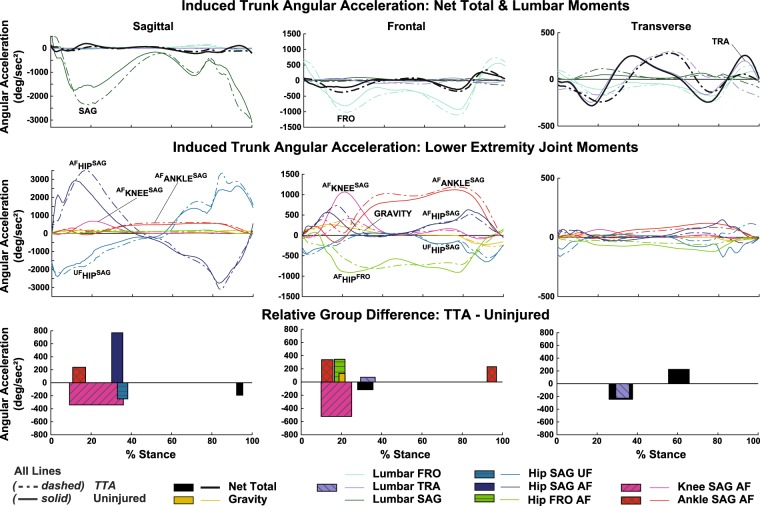
Ensemble average trunk angular acceleration trajectories induced by net joint moments and by gravity for the transtibial (TTA, dashed line) and uninjured group (solid line). Although all joint moments contribute to trunk accelerations, only the dominant trajectories for the lumbar (top row) and lower extremities (middle row) in each plane are shown for clarity. Significant between group differences are identified in duration by bar width and mean difference magnitude by bar height (bottom row). A positive difference indicates the TTA group had a more positive induced acceleration over the duration, relative to the reference uninjured group. AF = Affected limb, UF = Unaffected limb, SAG = Sagittal, FRO = Frontal, TRA = Transverse. Y-axis signage: Positive indicates trunk rotation towards the ipsilateral stance limb for frontal and transverse rotations, and anterior tilt for sagittal rotation.

Numerous differences in IA trajectories were identified between groups. A comprehensive accounting of timing and mean magnitude for each difference is provided in Supplementary Table S.1 and Fig. 3. The most notable differences were altered IAs corresponding to the affected limb ankle, knee, and hip moments during early stance in the sagittal and frontal planes. Compared to the uninjured group, the prosthetic ankle moment induced greater frontal ipsilateral IA (9–16%ST, *P* = 0.003; 92–97%ST, *P* = 0.007), and greater sagittal trunk flexion IA (11–17%ST, *P* = 0.002). The affected limb knee moment induced lesser sagittal trunk flexion IA (9–36%ST, *P* < 0.001) and lesser frontal ipsilateral IA (10–25%ST, *P* < 0.001). The affected limb hip extensor moment induced greater sagittal trunk flexion IA (30–36%ST, *P* = 0.016), while the unaffected limb hip flexor moment induced lesser trunk flexion IA (33–38%ST, *P* = 0.009). Lastly, the transverse lumbar moment induced lesser ipsilateral IA in the transverse plane (29–36%ST, *P* = 0.013) and greater ipsilateral IA in the frontal plane (30–37%ST, *P* = 0.009).

## Discussion and Conclusions

The aim of this study was to characterize how compensations in lower extremity joint moments contribute to altered, trunk angular dynamics in persons with a unilateral TTA during walking. An IA analysis was used to decompose trunk segment angular accelerations measured experimentally into constituent accelerations corresponding to net joint moments in the lumbar and lower extremities. As expected, there were differences in the magnitudes of net joint moments in persons with a TTA relative to uninjured persons that agreed with prior observational studies^8,10,11,13^, most notably: lesser knee extensor, hip extensor, and hip abductor moment magnitudes on the affected limb during early stance. Our hypothesis that trunk angular accelerations induced by net joint moments would differ between groups was supported for some – but not all – joints during specific durations of stance. In the frontal and sagittal plane during early stance, the TTA group had greater trunk IA from the prosthetic ankle, and lesser trunk IA from the affected limb knee. Additionally, the TTA group had brief durations of greater, sagittal trunk IA from the hip extensor moment and lesser, frontal trunk IA from the hip abductor moment relative to the uninjured group. Of note, not every trunk IA difference appeared to correspond with a concurrent difference in moment magnitude (e.g. early stance prosthetic ankle moment). This observation highlights that factors other than joint moment magnitude can influence the accelerations induced by a moment throughout the body – namely instantaneous positioning and velocity of all body segments relative to the joint moment.

### Ankle

In comparison to the uninjured ankle moment, the stance limb prosthetic ankle moment induced greater, ipsilaterally-directed angular accelerations on the trunk in the frontal plane. Prior IA analyses of uninjured persons walking found that during early stance the net ankle moment acts to rotate the trunk ipsilaterally over the stance limb in the frontal plane and tilt the trunk anteriorly in the sagittal plane^6^. We observed the same ankle IA mechanisms in this study, with greater magnitude in the TTA group relative to uninjured during 11–17%ST in the frontal plane and 9–16%ST in the sagittal plane (Fig. 3). Despite differences in trunk IA, the stance limb ankle moment magnitude was similar between groups during early stance. Consideration of the dynamic equations of motion aids interpretation of this and other IA findings:

1 $$\ddot{{\rm{q}}}=[{{\bf{M}}}^{-1}]\{{\bf{V}}({\rm{q}},{\dot{{\rm{q}}}}^{2})+{\bf{G}}({\rm{q}})+{\bf{F}}({\rm{q}},\dot{{\rm{q}}})+{\bf{T}}\}$$

where (${\rm{q}},\dot{{\rm{q}}},\ddot{{\rm{q}}})\,\,$are generalized coordinate positions, velocities, and accelerations. The inertial matrix **M** is a function of segmental moments of inertia and all instantaneous segment positions/orientations^20^. Eq. 1 illustrates that centrifugal velocity forces **V**, external gravitational forces **G**, kinematic ground constraint forces **F**, and net joint moments **T**, induce coordinate accelerations $\ddot{{\rm{q}}}$ throughout the whole body via the inverted system inertial matrix M^−1^ ^20,21^. Thus, for any given joint moment, the combination of instantaneous body posture and moment magnitude determine resultant accelerations induced on any given body segment. Applying this paradigm to our finding of greater trunk IA from the prosthetic ankle in the TTA group during early stance, Eq. 1 suggests that differences in body postural factors rather than joint moment magnitude primarily explain the altered trunk IA. In contrast, during late stance when persons with TTA had a greater ankle plantarflexor moment (Fig. 2), concurrently with greater frontal, ipsilateral trunk IA (Fig. 3), Eq. 1 suggests the altered IA was partly due to greater moment magnitude.

A prior simulation study of persons with unilateral TTA compared passive ankle-foot prosthesis function during walking to soleus and gastrocnemius functions in uninjured persons^22^. Findings suggested that the prosthesis did not fully replace plantarflexor muscle function for the sub-tasks of body propulsion and medio-lateral balance. Our findings supplement these observations, suggesting that the prosthetic ankle moment also has potential to destabilize frontal and sagittal trunk segment rotations in persons with TTA.

### Knee & hip abductors

In both groups, during early stance, the stance limb sagittal knee moment produced ipsilateral trunk IA in the frontal plane and trunk flexion IA in the sagittal plane, in agreement with prior IA studies^5,6^. In the TTA group, these mechanisms were diminished in both the frontal and sagittal planes (Fig. 3), likely driven by the lesser magnitude of knee extensor moment during 10–34%ST (Fig. 2). Deficits in knee extensor moment on the affected limb in early stance are commonly observed in comparisons of unilateral TTA and uninjured walking^8,9,23^. Our analyses show that in addition to knee-specific mechanical implications, a deficit in net knee moment can indirectly challenge frontal and sagittal control of trunk motion for persons with a unilateral TTA.

Even though there was substantially less frontal, ipsilateral trunk IA from the knee in the TTA group, net total frontal trunk acceleration was generally similar between groups. When summed, the following compensations  in the TTA group appear to yield similar net total trunk acceleration (i.e. for all but 28–36%ST, Fig. 3): greater ipsilateral IA from the prosthetic ankle and from gravity, lesser contralateral IA from the affected limb hip abductor moment, and greater contralateral IA from the frontal lumbar moment. This combination may represent an altered, whole body functional strategy to modulate lateral lean. Of note, the brief duration of greater net total contralateral acceleration during 28–36%ST *decelerated* greater frontal, ipsilaterally-directed velocity in the TTA group (Fig. 1). Subsequently, frontal ipsilateral lean angle was only marginally greater, by 2 degrees on average, in the TTA group across 22–44%ST (Supplementary Table S.1).

Prior observational studies of TTA walking have reported similar magnitudes of greater ipsilateral trunk lean over the prosthesis during midstance^1^. Hip abductor muscular strength deficits are often proposed as a causative mechanism of abnormal trunk lean^1,10^, although direct clinical evidence of an association is lacking. Our analysis showed the TTA group had a lesser affected limb hip abductor moment magnitude during 16–20%ST (Fig. 2), that occurred concurrently with lesser contralaterally-directed trunk IA from the hip abductor moment (Fig. 3). While conclusions cannot be drawn at the muscle group level via our net moment analysis, our results suggest that a decreased net hip abductor moment is associated with lesser deceleration of ipsilateral trunk lean in persons with a unilateral TTA.

### Hip flexors/extensors

In both groups, during the first half of stance, the affected limb hip extensor moment induced trunk flexion IA opposed by a similar magnitude of trunk extension IA from the unaffected hip flexor and sagittal lumbar moments, comparable to prior reports of uninjured persons walking^6^. Trunk flexion IA from the affected limb hip extensor was briefly greater in the TTA group (Fig. 3), which appeared driven by a concurrently increased extensor moment magnitude during 28–36%ST (Fig. 2). However, similar to the frontal plane, no differences in sagittal, experimentally-measured trunk angular acceleration occurred during this duration (Fig. 1), possibly due to greater extension-directed IA from the sagittal lumbar moment (Fig. 3). Greater hip extensor moments and power on the affected limb in early stance have been reported in observational comparisons of TTA and uninjured walking^11,24^. While likely employed as a compensatory mechanism to propel the body forward in the absence of active ankle plantarflexor power^11^, the compensation also appears to indirectly challenge modulation of sagittal trunk motion and increase demand on lumbar extensors.

### Transverse lumbar moment

In the transverse plane, the trunk angular acceleration trajectory in the TTA group generally lagged behind the uninjured group as highlighted by a delayed mid-stance peak in ipsilateral acceleration (Fig. 1). The result was greater, contralateral angular velocity during midstance acting to more vigorously twist the trunk away from the stance limb (Fig. 1). In both groups, the pattern of net total acceleration appears to be driven by the IA from the transverse lumbar moment in overall magnitude and timing patterns throughout stance, suggesting that control of transverse trunk motion is dominated by the lumbar, versus lower extremity joints, during walking (Fig. 3). This is in contrast to the sagittal and frontal planes, where lower extremity joints had important contributions alongside the respective lumbar moments.

### Limitations

While results of this study yield deeper insight into whole body control of trunk rotations in persons with a unilateral TTA, some limitations must be considered. Firstly, IAs of individual muscles, particularly those with bi-articular action, cannot be inferred from a net moment IA analysis and would instead require a muscle-driven dynamic model with simulated activations verified against experimental electromyography, as in Klemetti *et al*.^6^. Additionally, our results may not be generalizable to substantially different walking speeds from ~1.4m/sec, as joint moment compensations are known to increase with walking speed in persons with a unilateral TTA^11^, which may further alter trunk IAs. Lastly, some caution is necessary to avoid mis-representation of induced accelerations. While the analysis estimates IAs by applying each moment in isolation, IA trajectories should not be interpreted in isolation as physical accelerations. Rather, the theoretical function of each IA must be interpreted in the context of all other simultaneous IAs, which cumulatively equal the actual, experimentally measured motion.

### Clinical relevance

Results from this study are useful to guide follow-up work aimed at developing novel clinical assessments and interventions to stabilize trunk motion in patients with LEA. For example, a typical, visual gait assessment can only roughly assess normality of trunk kinematics (angles, velocities). The IA findings highlight this approach may overlook potentially deleterious mechanics (whole body force-acceleration couplings) which have potential to destabilize trunk kinematics. Secondly, physical therapy for trunk deviations generally focuses on the trunk-pelvis region with targeted closed kinetic chain strengthening of the core and hip abductors^25^. While this is sensible, our results suggest that prosthetic ankle and affected limb knee mechanics may also challenge sagittal and frontal control of trunk motion. Thus, movement training to modify position of lower extremity segments relative to the trunk (e.g. total limb ab/adduction or flexion), or adjustments to prosthetic ankle-foot mechanical properties (e.g., stiffness, active power, alignment), may be useful supplements for clinicians aiming to modify trunk motion in patients with a unilateral TTA.

### Conclusions

In conclusion, this study showed that persons with a unilateral TTA control trunk angular dynamics differently during walking in comparison to uninjured controls. Our primary finding is that the prosthetic ankle and affected limb knee impart different accelerations on the trunk in both the frontal and sagittal planes. Future dynamic simulations should investigate these trends at a whole body muscular level, while experimental studies should explore the effectiveness of dynamic, postural modifications and prosthetic ankle-foot device adjustments to address trunk movement deviations.

## Supplementary information

Supplementary Material

## Data Availability

The data analyzed and simulation setup files for the current study are available from the corresponding author on reasonable request.
