# Author Correction: Lower Extremity Joint Contributions to Trunk Control During Walking in Persons with Transtibial Amputation

**DOI:** 10.1038/s41598-019-53340-w

**Published:** 2019-11-13

**Authors:** Adam J. Yoder, Amy Silder, Shawn Farrokhi, Christopher L. Dearth, Brad D. Hendershot

**Affiliations:** 1 DoD-VA Extremity Trauma and Amputation Center of Excellence, Various locations, USA; 2 Department of Physical & Occupational Therapy, Naval Medical Center, San Diego, CA USA; 3 Department of Rehabilitation, Walter Reed National Military Medical Center, Bethesda, MD USA; 4 Department of Surgery, Uniformed Services University of the Health Sciences, Bethesda, MD USA; 5 Department of Rehabilitation Medicine, Uniformed Services University of the Health Sciences, Bethesda, MD USA

Correction to: *Scientific Reports* 10.1038/s41598-019-47796-z, published online 22 August 2019

The original version of this Article contained an error in the spelling of the name of the author Amy Silder, which was incorrectly given as Amy B. Silder. This has now been corrected in the PDF and HTML versions of the Article, and in the accompanying Supplementary Material file.

