# Supplementary material for: Lower Extremity Joint Contributions to Trunk Control During Walking in Persons with Transtibial Amputation
Source: Sci Rep. 2019 Aug 22;9:12267. doi: 10.1038/s41598-019-47796-z (PMC6706581; doi:10.1038/s41598-019-47796-z)
Supplement: Supplementary file 1 — Supplementary Material [file 41598_2019_47796_MOESM1_ESM.docx]

Lower Extremity Joint Contributions to Trunk Control During Walking in Persons with Transtibial Amputation

Adam J. Yoder^1,2^, Amy Silder^1,2^, Shawn Farrokhi^1,2^, Christopher L. Dearth^1,3,4^, Brad D. Hendershot^1,3,5^

## Supplementary Material


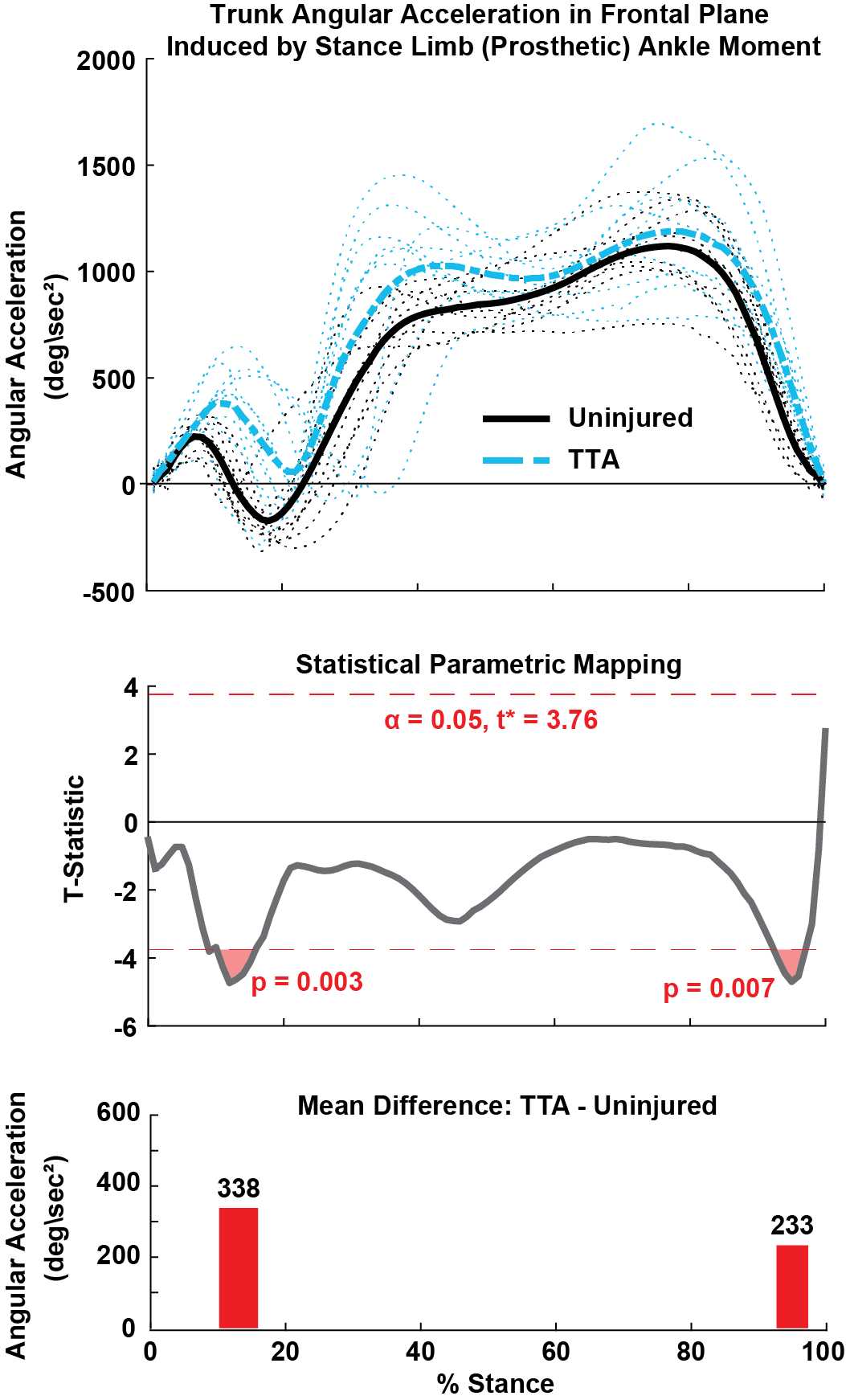


Fig. S.1 – Representative output for an un-paired t-test using statistical parametric mapping (SPM). Thick lines represent ensemble sample average and thin dotted lines distinguish individual subjects’ data for the uninjured and transtibial amputation (TTA) group. In this example, SPM identified two time durations where the stance limb ankle moment induced a greater angular acceleration on the trunk segment in the frontal plane in persons with a TTA: during 10-16% stance and 92-97% stance, with mean difference magnitudes of +338 deg/sec^2^ and +233 deg/sec^2^ (*P*=0.003, 0.007), indicating acceleration was directed more ipsilaterally in TTA persons relative to uninjured persons.

Table S.1 - Time duration (% stance) and magnitude (mean relative difference) of significantly different trajectories for joint moments, angular accelerations induced on the trunk, and the experimentally measured trunk angular acceleration, velocity, and angle. For each duration of stance over which group trajectories were significantly different, the magnitudes of differences are summarized as the transtibial amputation (TTA) group mean minus the uninjured subject group mean. AF=Affected, UF=Unaffected. SAG=Sagittal, FRO=Frontal, TRA=Transverse.

|  | % Stance | | Mean Difference  (TTA-Uninjured) | *P*-value |
| --- | --- | --- | --- | --- |
| **Joint Moment (N-m/kg)** | | | | |
| Hip _AF,SAG_ | 2 | 4 | 0.41 | 0.036 |
|  | 28 | 36 | -0.21 | 0.003 |
| Knee _AF,SAG_ | 2 | 4 | 0.19 | 0.041 |
|  | 10 | 34 | -0.44 | <0.001 |
| Hip _AF,TRA_ | 14 | 26 | -0.09 | <0.001 |
| Hip _UF,SAG_ | 22 | 24 | 0.14 | 0.041 |
| Hip _AF,FRO_ | 16 | 20 | 0.25 | 0.017 |
| Ankle _AF,SAG_ | 92 | 98 | -0.27 | 0.002 |
| **Trunk-Lab Angular Kinematics** | | | | |
| **Frontal** | | | | |
| Velocity (deg/s) | 14 | 17 | 11 | 0.045 |
| Angle (deg) | 22 | 44 | 2 | 0.032 |
| **Transverse** | | | | |
| Velocity (deg/s) | 33 | 55 | -21 | <0.001 |
| **Induced Angular Acceleration (Deg/sec^2^)** | | | | |
| **Frontal** | | | | |
| Knee _AF,SAG_ | 10 | 25 | -521 | <0.001 |
| Ankle _AF,SAG_ | 9 | 16 | 338 | 0.003 |
|  | 92 | 97 | 233 | 0.007 |
| Hip _AF,FRO_ | 16 | 22 | 344 | 0.011 |
| Lumbar _TRA_ | 30 | 37 | 71 | 0.009 |
| Gravity | 19 | 22 | 130 | 0.020 |
| Net Total | 28 | 36 | -117 | <0.001 |
| **Sagittal** | | | | |
| Knee _AF,SAG_ | 9 | 36 | -341 | <0.001 |
| Ankle _AF,SAG_ | 11 | 17 | 237 | 0.002 |
| Hip _AF,SAG_ | 30 | 36 | 769 | 0.016 |
| Hip _UF,SAG_ | 33 | 38 | -248 | 0.009 |
| Net Total | 92 | 95 | -192 | 0.025 |
| **Transverse** | | | | |
| Lumbar _TRA_ | 29 | 36 | -228 | 0.013 |
| Net Total | 26 | 38 | -245 | 0.000 |
|  | 55 | 66 | 225 | <0.001 |

Table S.2 – Tracking differences between modeled body markers and experimental markers after inverse kinematics. RMS=Root Mean Square

| Trunk (cm)  (C7 vertebrae, sternal notch, xiphoid, T8/T10 vertebrae) | | All Others (cm) | |
| --- | --- | --- | --- |
| Max | RMS | Max | RMS |
| 1.0(0.3) | 0.8(0.3) | 1.6(0.5) | 1.1(0.4) |

* Recommended quality standard is <4.0cm at maximum and <~2.0cm in RMS

Table S.3 – Magnitude of residual forces and moments between model and laboratory required to satisfy dynamic equilibrium. Values are after iterations of kinematic adjustment by residual reduction algorithm to improve agreement. Summarized as root-mean-squared average and absolute maximum over four quarters of stance. Magnitudes are normalized to % of maximum measured external ground reaction force. Recommended quality standards are <5.0% for forces and <1.0% for moments ^18^. F = Force, M=Moment, A/P=Anterior/Posterior, M/L=Medial/Lateral. DS1=Initial double support, ESS=Early single-stance, LSS=Late single-stance, DS2=Final Double Support

|  | | Residual Force (%Max Force) | | | | | | | |
| --- | --- | --- | --- | --- | --- | --- | --- | --- | --- |
|  | | RMS | | | | | Max | | |
| Gait Phase | | F_A/P_ | | F_Vertical_ | | F_M/L_ | F_A/P_ | F_Vertical_ | F_M/L_ |
| DS1 | | 0.5(0.2) | | 0.5(0.2) | | 0.2(0.1) | 0.8(0.3) | 0.7(0.4) | 0.3(0.1) |
| ESS | | 0.3(0.2) | | 0.6(0.3) | | 0.2(0.1) | 0.8(0.3) | 0.4(0.2) | 0.3(0.2) |
| LSS | | 0.3(0.2) | | 0.4(0.2) | | 0.3(0.2) | 0.9(0.2) | 0.5(0.2) | 0.5(0.2) |
| DS2 | | 0.6(0.2) | | 0.5(0.2) | | 0.4(0.2) | 0.9(0.3) | 1(0.4) | 0.8(0.4) |
| **Average** | | **0.4(0.2)** | | **0.5(0.2)** | | **0.3(0.2)** | **0.8(0.3)** | **0.7(0.4)** | **0.5(0.3)** |
|  | Residual Moment (%Max Force-Ht) | | | | | | | | |
|  | RMS | | | | | | Max | | |
| Gait Sub-phase | M_A/P_ | | M_Vertical_ | | M_M/L_ | | M_A/P_ | M_Vertical_ | M_M/L_ |
| DS1 | 0.2(0.1) | | 0.4(0.1) | | 0.2(0.1) | | 0.3(0.1) | 0.7(0.2) | 0.4(0.2) |
| ESS | 0.2(0.1) | | 0.2(0.1) | | 0.3(0.1) | | 0.3(0.1) | 0.3(0.1) | 0.4(0.1) |
| LSS | 0.3(0.2) | | 0.4(0.2) | | 0.2(0.1) | | 0.4(0.3) | 1.0(0.4) | 0.5(0.2) |
| DS2 | 0.4(0.2) | | 0.6(0.2) | | 0.4(0.2) | | 0.6(0.3) | 1.1(0.3) | 0.6(0.3) |
| **Average** | **0.3(0.2)** | | **0.4(0.2)** | | **0.3(0.1)** | | **0.4(0.3)** | **0.8(0.4)** | **0.5(0.2)** |

Table S.4 - Root-mean-square average difference between experimental GRF and simulated GRF predicted by a rolling-without-slipping foot constraint over four quarters of stance, normalized by bodyweight and reported 0-100%. Values are sample mean (standard deviation). DS1=Initial double support, ESS=Early single-stance, LSS=Late single-stance, DS2=Final Double Support

| Stance Phase | M_Free_ | F_A/P_ | F_M/L_ | F_Vert_ |
| --- | --- | --- | --- | --- |
| DS1 | 0.2(0.1) | 1.1(0.3) | 1.9(0.7) | 3.9(1.1) |
| ESS | 0.0(0.0) | 0.1(0.0) | 0.1(0.1) | 0.4(0.2) |
| LSS | 0.0(0.0) | 0.3(0.2) | 0.3(0.2) | 0.3(0.2) |
| DS2 | 0.1(0.1) | 1.1(0.5) | 1.7(0.6) | 4.2(1.5) |
| **Stance Average** | **0.1(0.1)** | **0.7(0.6)** | **1.0(0.9)** | **2.2(2.8)** |

Table S.5 – Root-mean-square average difference between the net summation of induced trunk angular accelerations from the 11 joint moments, gravity, and velocity effects (simulated acceleration) and the experimentally measured trunk segment acceleration in global coordinates, within four quarters of stance. In each plane for each subject, the average simulated versus experimental RMS difference was normalized to the max induced acceleration across all system forces and reported 0-100%. Values are sample mean (standard deviation). DS1=Initial double support, ESS=Early single-stance, LSS=Late single-stance, DS2=Final Double Support

| Stance Phase | $(Normalized \% Difference)=\frac{RMS Average Acceleration Difference}{Maximum Induced Acceleration}\times100\%$ | | |
| --- | --- | --- | --- |
|  | Frontal | Sagittal | Transverse |
| DS1 | 11(3)% | 3(1)% | 6(4)% |
| ESS | 1(1)% | < 1(0)% | < 1(0)% |
| LSS | 1(1)% | < 1(0)% | < 1(0)% |
| DS2 | 11(4)% | 3(2)% | 4(3)% |
| **Stance Average** | **6(6)%** | **2(2)%** | **3(4)%** |
